# Supplementary material for: Helminth exposure and immune response to the two-dose heterologous Ad26.ZEBOV, MVA-BN-Filo Ebola vaccine regimen
Source: PLoS Negl Trop Dis. 2024 Apr 11;18(4):e0011500. doi: 10.1371/journal.pntd.0011500 (PMC11037528; doi:10.1371/journal.pntd.0011500)
Supplement: S1 Appendix — Table A. Description of the helminth ELISA commercial kits used, measured parasitic infection and cross reactivity. Table B. Overview of the substudy available sample selection by study (EBL2001, EBL2002, EBL3001), age group and country. Table C. Helminth ELISA test results at baseline among all participants of the helminth substudy (EBL2001, EBL2002, EBL3001 pooled). Table D. Characteristics of participants (pooled EBL2001, EBL2002, 3001) by helminth ELISA test result. Table E. Description of helminth ELISA test results in pooled EBL2001, EBL2002 and EBL3001 studies. Table F. Description of eosinophil levels among participants from studies EBL2001, EBL2002 and EBL3001. Table G. Description of eosinophil levels by any helminth ELISA test among participants from studies EBL2001 and EBL2002. Table H. Description of eosinophil levels in participants with any Helminth positive ELISA test from EBL2001 and EBL2002 studies (N = 55). Fig A. Description of the EBOV-GP antibodies at 21 days post dose 2 by eosinophil count. Fig B. Correlation between EBOV GP binding antibody geometric mean concentrations (GMC) and inflammatory markers. Fig C. Inflammatory markers (pg/mL) measured at baseline significantly associated with helminth test positivity (0 = No positive helminth ELISA test; 1 = any positive helminth ELISA test). Fig D. Inflammatory markers (pg/mL) statistically different between studies (EBL2001, EBL2002, EBL3001) at baseline. (DOCX) [file pntd.0011500.s001.docx]

SUPPLEMENTARY MATERIALS

Summary table

[Section A: Helminth ELISA commercial kits 2](#_Toc161667806)

[Table A. Description of the helminth ELISA commercial kits used, measured parasitic infection and cross reactivity 2](#_Toc161667807)

[Section B: Sample Size Calculation 2](#_Toc161667808)

[Text A. Description of Sample Size calculation 2](#_Toc161667809)

[Table B. Overview of the substudy available sample selection by study (EBL2001, EBL2002, EBL3001), age group and country. 3](#_Toc161667810)

[Section C: Description of helminth ELISA test results in the population 3](#_Toc161667811)

[Table C: Helminth ELISA test results at baseline among all participants of the helminth substudy (EBL2001, EBL2002, EBL3001 pooled). 3](#_Toc161667812)

[Table D: Characteristics of participants (pooled EBL2001, EBL2002, 3001) by helminth ELISA test result 4](#_Toc161667813)

[Table E: Description of helminth ELISA test results in pooled EBL2001, EBL2002 and EBL3001 studies 5](#_Toc161667814)

[Section D: Eosinophil levels at baseline 6](#_Toc161667815)

[Table F: Description of eosinophil levels among participants from studies EBL2001, EBL2002 and EBL3001 6](#_Toc161667816)

[Table G: Description of eosinophil levels by any helminth ELISA test among participants from studies EBL2001 and EBL2002 6](#_Toc161667817)

[Table H: Description of eosinophil levels in participants with any Helminth positive ELISA test from EBL2001 and EBL2002 studies (N= 55) 6](#_Toc161667818)

[Fig A: Description of the EBOV-GP binding antibodies concentration 21 days post dose 2 by eosinophil count 7](#_Toc161667819)

[Section E: Inflammatory markers 8](#_Toc161667820)

[Fig B: Correlation between EBOV GP binding antibody geometric mean concentrations (GMC) and inflammatory markers 21 days post dose 2 and 365 days post dose 1. 9](#_Toc161667821)

[Fig C. Inflammatory markers (pg/mL) measured at baseline significantly associated with helminth test positivity (0 = No positive helminth ELISA test; 1 = any positive helminth ELISA test). 10](#_Toc161667822)

[Fig D. Inflammatory markers (pg/mL) statistically different between studies (EBL2001, EBL2002, EBL3001) at baseline. 18](#_Toc161667823)

# Section A: Helminth ELISA commercial kits

### Table A. Description of the helminth ELISA commercial kits used, measured parasitic infection and cross reactivity

| **Parasitic infection** | **Serology kit** | **Cross reactivity** |
| --- | --- | --- |
| **Schistosomiasis**  *Mansoni and haematobium* | ELISA *Schistosoma mansoni,*  Bordier (#9600) | Filariasis and leishmaniasis |
| **Filariasis**  *Onchocerca volvili*  Lymphatic filariasis  *Loa loa* | ELISA *Acanthocheilonema viteae,* Bordier (#9400) | 40-83% with trichinellosis, hydatidosis, fascioliasis, ankylostomiasis and strongyloidiasis |
| **Strongyloidiasis** | ELISA *Strongyloïdes ratti,*  Bordier (#9450) | With shistosomiasis, filariasis, toxocariasis, fascioliasis and amebiasis |

# Section B: Sample Size Calculation

### Text A. Description of Sample Size calculation

As EBL2002 enrolled three groups (different intervals between vaccinations) in the healthy adults’ cohorts from four countries, a total of 93 (28+24+41) participants was necessary. Therefore, 24 samples were retrieved randomly from each country. Two groups (0, 28 and 0, 56 regimens) were tested in the adolescent and children cohorts giving a total of 52 participants needed (28 and 24 participants, respectively). As there was one vaccination regimen (0, 56 interval) tested in the EBL3001 study, 24 participants’ samples were needed in each cohort (healthy adults, adolescents, children and toddlers). In the EBL2001 study, the three vaccination regimens were tested in 2 countries. A total of 92 samples was necessary (46 participant samples from each country). However, due to unavailability of samples from one country, the required number was taken from the other country in the EBL2001 study. The minimum sample size required, from all countries was 385 (197 in EBL2002, 96 in EBL3001 and 92 in EBL2002) but we retrieved 388 samples due to the 93 adults’ samples to be divided into 4 countries. We ended up by taking 24 samples from each country.

### Table B. Overview of the substudy available sample selection by study (EBL2001, EBL2002, EBL3001), age group and country.

|  | **EBL2001** | | **EBL2002** | | | | **EBL3001** |  |
| --- | --- | --- | --- | --- | --- | --- | --- | --- |
| Age Group-Country | France | UK | Burkina Faso | Cote d’Ivoire | Kenya | Uganda | **Sierra Leone** | **Total** |
| Healthy adults | **36** | **56** | **24** | **24** | **24** | **24** | **24** | **212** |
| Adolescents (cohort 2b) | **_** | **_** | **18** | **-** | **17** | **16** | **24** | **75** |
| Children (cohort 3) | **_** | **_** | **14** | **18** | **8** | **13** | **24** | **77** |
| Children 1-3 |  |  |  |  |  |  | **24** | **24** |
| **Total** | **36** | **56** | **56** | **42** | **49** | **53** | **96** | **388** |
| **Total study** | **92** | | **200** | | | | **96** |  |

# Section C: Description of helminth ELISA test results in the population

### Table C: Helminth ELISA test results at baseline among all participants of the helminth substudy (EBL2001, EBL2002, EBL3001 pooled).

| Presence of helminth IgG | No | Yes | Total |
| --- | --- | --- | --- |
| Any helminth | 289 (78.7%) | 78 (21.3%) | 367 (100%) |
| *S. mansoni* | 327 (89.1%) | 40 (10.9) | 367 (100%) |
| *A. viteae* | 334 (91%) | 33 (9%) | 367 (100%) |
| *S. ratti* | 338 (92.1%) | 29 (7.9%) | 367 (100%) |
| *S. mansoni or A. viteae* | 303 (82.6%) | 64 (17.4%) | 367 (100%) |
| *S. mansoni or S. ratti* | 306 (63.4%) | 61 (16.6%) | 367 (100%) |
| *S. ratti or A. viteae* | 316 (86.1%) | 51 (13.9%) | 367 (100%) |
| *S. mansoni & A. viteae* | 358 (97.5%) | 9 (2.5%) | 367 (100%) |
| *S. mansoni & S. ratti* | 359 (97.8%) | 8 (2.2%) | 367 (100%) |
| *S. ratti & A. viteae* | 356 (97%) | 11 (3%) | 367 (100%) |
| *S. mansoni & A. viteae & S. ratti* | 363 (98.9%) | 4 (1.1%) | 367 (100%) |

### Table D: Characteristics of participants (pooled EBL2001, EBL2002, 3001) by helminth ELISA test result

| Characteristic | **Presence of any helminth IgG** | | **Overall**  (N = 367)*^1^* | **p-value***^2^* |
| --- | --- | --- | --- | --- |
|  | **No**  (N = 289)*^1^* | **Yes**  (N = 78)*^1^* |  |  |
| **Age group** |  |  |  | **0.054** |
| Children | 88 (85%) | 15 (15%) | 103 (100%) |  |
| Adolescents | 59 (82%) | 13 (18%) | 72 (100%) |  |
| Adults | 142 (74%) | 50 (26%) | 192 (100%) |  |
| **Sex** |  |  |  | **0.20** |
| Female | 126 (75%) | 41 (25%) | 167 (100%) |  |
| Male | 163 (82%) | 37 (18%) | 200 (100%) |  |
| **Study** |  |  |  | **0.015** |
| EBL2001 | 78 (90%) | 9 (10%) | 87 (100%) |  |
| EBL2002 | 137 (74%) | 47 (26%) | 184 (100%) |  |
| EBL3001 | 74 (77%) | 22 (23%) | 96 (100%) |  |
| **Country** |  |  |  | **0.006** |
| France | 30 (88%) | 4 (12%) | 34 (100%) |  |
| UK | 48 (91%) | 5 (9.4%) | 53 (100%) |  |
| Burkina Faso | 41 (75%) | 14 (25%) | 55 (100%) |  |
| Cote d’Ivoire | 25 (68%) | 12 (32%) | 37 (100%) |  |
| Kenya | 38 (90%) | 4 (9.5%) | 42 (100%) |  |
| Uganda | 33 (66%) | 17 (34%) | 50 (100%) |  |
| Sierra Leone | 74 (77%) | 22 (23%) | 96 (100%) |  |
| *^1^*n (%)  *^2^*Pearson's Chi-squared test | | | | |

### Table E: Description of helminth ELISA test results in pooled EBL2001, EBL2002 and EBL3001 studies

| **Characteristic** | **None** (N = 289) | **Single positive helminth ELISA** | | | **Multiple positive helminth ELISAs** | | | **Overall**  (N = 367) |
| --- | --- | --- | --- | --- | --- | --- | --- | --- |
|  |  | ***A. viteae***  (N = 17) | ***S. mansoni***,  (N = 27) | ***S. ratti***,  (N = 14) | ***A.viteae + S. ratti***,  (N = 7) | ***S. mansoni+ A. viteae***,  (N = 5) | ***S. mansoni +S. ratti***,  (N = 8) |  |
| **Age group** |  |  |  |  |  |  |  |  |
| Children | 88 (30%) | 4 (24%) | 4 (15%) | 1 (7.1%) | 3 (43%) | 0 (0%) | 3 (38%) | 103 (28%) |
| Adolescents | 59 (20%) | 3 (18%) | 5 (19%) | 1 (7.1%) | 0 (0%) | 4 (80%) | 0 (0%) | 72 (20%) |
| Adults | 142 (49%) | 10 (59%) | 18 (67%) | 12 (86%) | 4 (57%) | 1 (20%) | 5 (62%) | 192 (52%) |
| **Sex** |  |  |  |  |  |  |  |  |
| F | 126 (44%) | 11 (65%) | 12 (44%) | 9 (64%) | 3 (43%) | 3 (60%) | 3 (38%) | 167 (46%) |
| M | 163 (56%) | 6 (35%) | 15 (56%) | 5 (36%) | 4 (57%) | 2 (40%) | 5 (62%) | 200 (54%) |
| **Race** |  |  |  |  |  |  |  |  |
| African | 218 (75%) | 16 (94%) | 26 (96%) | 9 (64%) | 7 (100%) | 5 (100%) | 8 (100%) | 289 (79%) |
| White or other | 71 (25%) | 1 (5.9%) | 1 (3.7%) | 5 (36%) | 0 (0%) | 0 (0%) | 0 (0%) | 78 (21%) |
| **Study** |  |  |  |  |  |  |  |  |
| EBL2001 | 78 (27%) | 1 (5.9%) | 1 (3.7%) | 6 (43%) | 0 (0%) | 0 (0%) | 1 (12%) | 87 (24%) |
| EBL2002 | 137 (47%) | 14 (82%) | 18 (67%) | 7 (50%) | 4 (57%) | 3 (60%) | 1 (12%) | 184 (50%) |
| EBL3001 | 74 (26%) | 2 (12%) | 8 (30%) | 1 (7.1%) | 3 (43%) | 2 (40%) | 6 (75%) | 96 (26%) |
| **Country** |  |  |  |  |  |  |  |  |
| France | 30 (10%) | 1 (5.9%) | 0 (0%) | 2 (14%) | 0 (0%) | 0 (0%) | 1 (12%) | 34 (9.3%) |
| UK | 48 (17%) | 0 (0%) | 1 (3.7%) | 4 (29%) | 0 (0%) | 0 (0%) | 0 (0%) | 53 (14%) |
| Burkina Faso | 41 (14%) | 2 (12%) | 10 (37%) | 0 (0%) | 0 (0%) | 2 (40%) | 0 (0%) | 55 (15%) |
| Cote d’Ivoire | 25 (8.7%) | 2 (12%) | 4 (15%) | 2 (14%) | 3 (43%) | 0 (0%) | 1 (12%) | 37 (10%) |
| Kenya | 38 (13%) | 0 (0%) | 2 (7.4%) | 2 (14%) | 0 (0%) | 0 (0%) | 0 (0%) | 42 (11%) |
| Uganda | 33 (11%) | 10 (59%) | 2 (7.4%) | 3 (21%) | 1 (14%) | 1 (20%) | 0 (0%) | 50 (14%) |
| Sierra Leone | 74 (26%) | 2 (12%) | 8 (30%) | 1 (7.1%) | 3 (43%) | 2 (40%) | 6 (75%) | 96 (26%) |

# Section D: Eosinophil levels at baseline

### Table F: Description of eosinophil levels among participants from studies EBL2001, EBL2002 and EBL3001

| **Eosinophil count (per mm3)** | **EBL2001**  (N = 87) | **EBL2002**  (N = 184) | **EBL3001**  (N = 96) | **Overall**  (N = 367) |
| --- | --- | --- | --- | --- |
| 0-500 | 85 (100%) | 165 (90%) | NA | 250 (93%) |
| > 500 | 0 (0%) | 18 (9.8%) | NA | 18 (6.7%) |
| Unknown | 2 | 1 | 96 | 99 |

### Table G: Description of eosinophil levels by any helminth ELISA test among participants from studies EBL2001 and EBL2002

| **Eosinophil count (per mm^3^)** | **Positive helminth ELISA test^1^** | | **All** |
| --- | --- | --- | --- |
|  | **No** | **Yes** |  |
| 0-500 (normal) | 204(95.8%) | 46(83.6%) | 250(93.3%) |
| > 500 (hyper-eosinophilia) | 9(4.2%) | 9(16.4%) | 18(6.7%) |
| Total | 213(100%) | 55(100%) | 268(100%) |

^1^ Positive helminth ELISA test: at least one positive helminth ELISA test

### Table H: Description of eosinophil levels in participants with any Helminth positive ELISA test from EBL2001 and EBL2002 studies (N= 55)

| **Eosinophil count (per mm3)** | **EBL2001** | **EBL2002** | **Overall** |
| --- | --- | --- | --- |
| 0-500 (normal) | 9 (100%) | 37 (80.4%) | 46 (83.6%) |
| > 500 (hyper-eosinophilia) | 0 (0%) | 9 (19.6%) | 9 (16.4%) |
| Overall | 9 (100%) | 46 (100%) | 55 (100%) |

### Fig A: Description of the EBOV-GP binding antibodies concentration 21 days post dose 2 by eosinophil count

# Section E: Inflammatory markers

**a. Day 21 post dose 2**

The dots represent the correlation between each inflammatory markers, EBOV GP GMC 21 days post dose 2 and age in x axis and each of the other’s markers, EBOV GP GMC 21 days post dose 2 and age on the y-axis.

On the graduated bar on the right, the blue color represents the negative correlation and the brown the positive correlation, more the color is dark, more the correlation is strong

**b. Day 365 post dose 1 vaccination**

The dots represent the correlation between each inflammatory markers, EBOV GP GMC 365 days post dose 1and age in x axis and each of the other’s markers, EBOV GP GMC 365 days post dose 1 and Age on the y-axis.

On the graduated bar on the right, the blue color represents the negative correlation and the brown the positive correlation, more the color is dark, more the correlation is strong.

For example, IL6 is strongly correlated to IL.1b.IL.1F2 and to CCL4.MIP.1b

### Fig B: Correlation between EBOV GP binding antibody geometric mean concentrations (GMC) and inflammatory markers 21 days post dose 2 and 365 days post dose 1.

p-value = 0.002 p-value = 0.002

p-value = 0.004 p-value= 0.008

p-value = 0.002

### Fig C. Inflammatory markers (pg/mL) measured at baseline significantly associated with helminth test positivity (0 = No positive helminth ELISA test; 1 = any positive helminth ELISA test).

p-value < 0.001 p-value < 0.001

p-value < 0.001 p-value < 0.001

p-value = 0.01 p-value = 0.05

p-value < 0.001 p-value = 0.02

p-value = 0.002 p-value = 0.005

p-value < 0.001 p-value = 0.003

p-value = 0.002 p-value < 0.001

p-value < 0.001 p-value < 0.001

p-value < 0.001 p-value < 0.001

p-value = 0.03 p-value < 0.001

p-value < 0.001 p-value < 0.001

p-value < 0.001 p-value < 0.001

p-value < 0.001 p-value = 0.03

p-value = 0.01 p-value = 0.007

p-value = 0.002 p-value < 0.001

p-value < 0.001 p-value < 0.001

### Fig D. Inflammatory markers (pg/mL) statistically different between studies (EBL2001, EBL2002, EBL3001) at baseline.
