# Supplementary material for: Helminth exposure and immune response to the two-dose heterologous Ad26.ZEBOV, MVA-BN-Filo Ebola vaccine regimen
Source: PLoS Negl Trop Dis. 2024 Apr 11;18(4):e0011500. doi: 10.1371/journal.pntd.0011500 (PMC11037528; doi:10.1371/journal.pntd.0011500)
Supplement: Attachment — Submitted filename: Helminth_Exposure_Rebuttal_Letter_PLOS_NTDs_R1_HB_05122023.pdf [file pntd.0011500.s002.pdf]

4th December 2023

Object: Revision of manuscript: PNTD-D-23-00840

**Helminth exposure and immune response to the two-dose heterologous Ad26.ZEBOV, MVA-BN-Filo Ebola vaccine regimen**

Dear Eva Clark,

Thank you for sharing with us the editorial and reviewer's comments on the above-named manuscript. We are pleased that the reviewer believes that this is a well-written and a captivating topic, and we thank him for his insightful and helpful comments. We have taken the editorial and reviewer's comments into consideration and we have revised the manuscript. Please, find below our response to the specific comments.

We hope that the paper is now acceptable for publication in *PLOS Neglected Tropical Diseases*.

Yours sincerely,

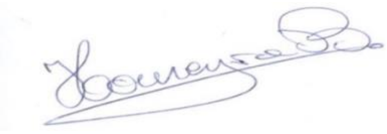

Houreratou BARRY

On behalf of all co-authors

**Response to reviewer comments:**

## Methods

Reviewer #1:

- The authors have adeptly delineated both the primary and secondary objectives of the study.

- The study design demonstrates a high level of validity.

- The description of the study population is lucid and comprehensive.

- The calculated sample size aligns well with the scope of the study.

- The utilization of appropriate statistical analyses enhances the rigor of the study.

1. However, it's important to note that while the authors state using RStudio for data analysis, it's worth clarifying that R programming was the actual tool employed. RStudio serves as a user-friendly interface for R programming and should be appropriately distinguished from the underlying analytical language. (Line 250, page9)

> ***Response: thank you for the comment, this point has been corrected***
>
> ***Text corrected under statistical analysis:*** *All analyses were conducted using R version 1.2.5033 and a p-value ≤ 0.05 was considered as statistically significant.*

2. On line 182, page 6, it was mentioned that participants from each initial study were randomly chosen. Could you please clarify if the number from each study was taken in proportion, or if all participants were combined and then selected randomly? This clarification would help readers better understand the process.

> ***Response: Thanks for the comment. We have added some text for the selection process in study design section to clarify (see below)***
>
> ***Text added under heading 'Study design':*** *The samples of Participants included in the present study were randomly selected from remaining samples of the initial clinical trials separately for each study, study interval group, age group and country. This was based on the calculated sample size (Appendix 2).*

3. Addressing a minor concern, it remains unclear if this study is an extension of the previously mentioned clinical trials, thus potentially obviating the need for new ethical approval. It would be beneficial for the authors to elaborate in more explicit terms on how they ensured adherence to ethical requirements in light of this potential continuity with previous trials.

***Response: We confirm that this study is an ancillary study to the previously mentioned clinical trials. As such, initial frozen serum samples were retrieved and analysed.***
***The study was validated by the scientific board and the sponsor of the main study and the initial informed consent obtained for the main study covered the use of the samples for other scientific research related to the vaccine and Ebola virus disease.***

***Text added under study design section:*** *All adult participants and parents/legal guardian of adolescents and children supplied written informed consent before enrolment in the main trials. Adolescent and older children also provided written informed ascent before inclusion. Informed consent from the main trials stated the use of participants information and samples for the study and other scientific research related to the vaccine and to Ebola virus disease.*

**Results**

Reviewer #1: - The executed analyses are consistent with the authors' outlined methodology.
- The presentation of results is notably clear and comprehensible.
- Figure 1 is easy to comprehend, but its quality seems to be suboptimal. It might be beneficial to enlarge the numbers within the figure, as they appear quite small at present.

***Response: Thank you for the comment, the quality of Figure 1 has been improved***

**Conclusions**

Reviewer #1: - The conclusions drawn align logically with the presented data.

- The limitations of the study are distinctly articulated.

- Given that the study's outcomes indicate an absence of linkage between immune markers of helminth exposure and post-vaccination response, it would be advantageous for the authors to delve into potential factors contributing to the varying immunogenicity of the Ebola vaccine across diverse regions. This could serve to capture readers' attention and foster future research initiatives.

***Response: Thanks for the comment. We have added more discussion about other potential factors (lines 386-404,422-428 page17 and 434-439on page 18)***

***Text added under discussion section:*** *Although no clear association between EBOV-GP antibody concentration post Ad26.ZEBOV, MVA -BN-Filo vaccination and exposure to the three helminths tested was found, other helminth could be associated with the immune response. Indeed, specific helminth-derived products have been characterized as having immunomodulatory effects, including lysophosphatidylserine, phosphatidylserine, lacto-N-fucopentaose III, Acanthocheilonema viteae ES62, Schistosoma mansoni dsRNA, cathepsin cysteine proteases and others. These products may have effects that function via interactions with Toll-like receptors (TLR), including TLR-2,3, or 4 in particular, or may impact TLR signaling pathways. Certain immunomodulatory effects may also function via TLR-independent mechanisms [21].*
*In Africa the major soil-transmitted helminth (STH) infections; hookworm (Necator americanus and Ancylostoma duodenale, large roundworms (Ascaris lumbricoides), and*

*whipworms (Trichuris trichiura) are often co-endemic with Schistosoma mansoni, a trematode parasite [25]. These three helminth tests were chosen in our study for two other reasons in addition to their documented immunomodulatory effect. The first reason was their high prevalence in some of our target countries and the second reason was the availability of the tests. In Sierra Leone, a national school-based survey conducted in 2012 and 2016 to evaluate the progress of Mass Drug Administration against helminth infections found an overall Schistosoma mansoni prevalence in the seven MDA districts of 20.4% (95% CI: 18.7–22.3%) [33]. In the "haut Bassin region" of Burkina Faso, this was 8.75% [35].*

**Text added under discussion section:** *In conclusion, the results of the present study do not suggest an association between the immune response to helminths exposure and the response to the Ad26.ZEBOV, MVA-BN-Filo vaccine regimen. Nonetheless, several studies have reported lower vaccine immunogenicity, in developing countries for some vaccines [43- 45]. Various hypotheses explaining variability of the immune response have been made, notably genetic factors, environmental factors such as activated immune microenvironment, exposure to environmental pathogens (bacterial, viral, parasitic) microbiome, pre-existing immunity specially for adenovirus and demographic factors such as age, sex and BMI. All these factors are potential sources for the variability of the Ad26.ZEBOV, MVA-BN-Filo Ebola vaccine-induced immune responses between countries.*
*Assessing for other parasitic infections using more advances technologies, serology of viral infections such as CMV and Direct measurement of the microbiota present at the time of vaccination may provide additional information on the potential factors associated with variation in the response to vaccines in Africa [46].*

.

**References**

***References number 35 on line 640 page 22 and numbers 43-45 on line 679-688 page 23 have been added to the reference list.***
